# Stable Na Electrodeposition Enabled by Agarose-Based Water-Soluble Sodium Ion Battery Separators

**DOI:** 10.1021/acsami.1c02135

**Published:** 2021-04-29

**Authors:** Alazne Ojanguren, Neeru Mittal, Erlantz Lizundia, Markus Niederberger

**Affiliations:** † Laboratory for Multifunctional Materials, Department of Materials, ETH Zürich, Vladimir-Prelog-Weg 5, 8093 Zurich, Switzerland; ‡ Life Cycle Thinking Group, Department of Graphic Design and Engineering Projects, Faculty of Engineering in Bilbao, University of the Basque Country (UPV/EHU), Bilbao 48013, Spain; § BCMaterials, Basque Center for Materials, Applications and Nanostructures, UPV/EHU Science Park, 48940 Leioa, Spain

**Keywords:** agarose, degradability, sodium ion battery (NIB), sodium plating/stripping, battery life span, circular economy

## Abstract

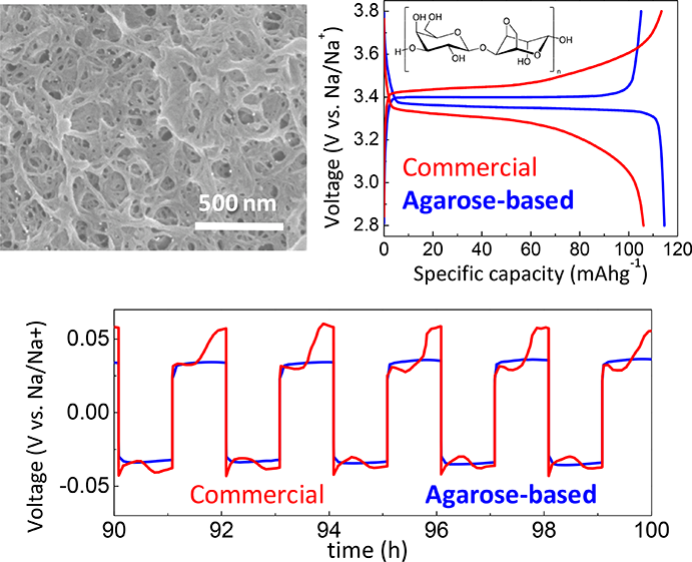

Developing efficient energy storage technologies is at the core of current strategies toward a decarbonized society. Energy storage systems based on renewable, nontoxic, and degradable materials represent a circular economy approach to address the environmental pollution issues associated with conventional batteries, that is, resource depletion and inadequate disposal. Here we tap into that prospect using a marine biopolymer together with a water-soluble polymer to develop sodium ion battery (NIB) separators. Mesoporous membranes comprising agarose, an algae-derived polysaccharide, and poly(vinyl alcohol) are synthesized via nonsolvent-induced phase separation. Obtained membranes outperform conventional nondegradable NIB separators in terms of thermal stability, electrolyte wettability, and Na^+^ conductivity. Thanks to the good interfacial adhesion with metallic Na promoted by the hydroxyl and ether functional groups of agarose, the separators enable a stable and homogeneous Na deposition with limited dendrite growth. As a result, membranes can operate at 200 μA cm^–2^, in contrast with Celgard and glass microfiber, which short circuit at 50 and 100 μA cm^–2^, respectively. When evaluated in Na_3_V_2_(PO_4_)_3_/Na half-cells, agarose-based separators deliver 108 mA h g^–1^ after 50 cycles at C/10, together with a remarkable rate capability. This work opens up new possibilities for the use of water-degradable separators, reducing the environmental burdens arising from the uncontrolled accumulation of electronic waste in marine or land environments.

## Introduction

Developing efficient, clean, and renewable energy conversion and storage technologies is one of the key targets for a sustainable society to counteract the depletion of natural resources, global warming, and environmental pollution.^1−3^ The so-called “energy transition” has a central role to play in climate change mitigation and aims to replace fossil energy production with renewable energy sources.^4,5^ The different policy approaches such as the European Green Deal, the Energy Efficiency and Consumer Engagement, or the EU Strategy for Greenhouse Gas Emissions Reductions are proof of this priority.^6^

Energy storage systems based on renewable and degradable materials may allow full exploitation of renewable energy sources and boost electromobility,^7^ while lowering the environmental footprint of conventional energy storage systems. This can be accomplished within a circular economy perspective according to “reuse, recycle, or biodegrade”, limiting resource depletion and smoothing end-of-life scenarios.^8^ In this context, electrochemical energy storage (EES) systems are of particular relevance as they can store and deliver on-demand power.^9^ Because of their high energy densities, low self-discharge rates, and long operation life spans, lithium ion batteries (LIBs) are one of the most mature batteries,^10^ overshadowing other relevant technologies. However, LIBs require scarce, costly, and toxic materials (lithium, cobalt, nickel, etc.) to operate, making their application challenging from the sustainability viewpoint.^11^

Conversely, sodium ion batteries (NIBs) rely on earth-abundant and nontoxic sodium ions (Na^+^) as charge carriers.^12^ NIBs are considered as the closest EES in both technology and chemistry to LIBs, making them good candidates to replace LIBs in the medium term. With a gravimetric energy density of sodium of 1165 Wh kg^–1^,^13^ current NIBs use separators based on polypropylene, polyethylene, poly(vinylidene fluoride), or glass microfiber. The widespread use of these nondegradable and nonrenewable materials threatens human and environmental safety as they generate undesired waste streams ending up directly into oceans or landfills,^1,14^ while accelerating the depletion of finite natural resources. In addition, these separators are hydrophobic (poorly wettable by electrolytes),^15^ too dense to enable efficient Na^+^ transport, and mechanically/thermally unstable, thus jeopardizing the battery safety.^16^

A solution to upgrade the performance and safety delivered by NIBs while reducing their environmental footprint could come from the replacement of conventional separators by membranes with enhanced functionalities based on renewable and degradable materials. As part of a circular economy strategy, degradable battery components would reduce the uncontrolled accumulation of electronic waste in the terrestrial or marine ecosystems while increasing material retrieval rates during recycling, thus lowering the environmental footprint associated with battery disposal.^8^ In this context, polysaccharides fulfill the often self-excluding requisites of good electrochemical performance, renewability, and degradability.^17,18^ Plant-based (cellulose^19^ and lignin)^20^ or animal-based polysaccharides (chitin)^21^ have already shown good potential as battery separators. However, nature provides plenty of fascinating long-chain polymeric carbohydrates which have not yet been fully exploited as battery separators, opening the door to the design of degradable batteries with enhanced performance and safety.

In comparison with many petroleum-based polymers that are only degradable under specific enzymatic and catalytic conditions,^22,23^ the marine-based polysaccharide agarose is an easily degradable material with suitable functionalities for battery applications. Commonly used in the food, pharmaceutical, and biomedical industries, agarose presents a neutral linear structure composed of alternating d-galactose and 3,6-anhydro-l-galactose units linked by alternating β-4 → 1 and α-1 → 3 bonds.^24^ Agarose can be easily processed in hot water with no need of harsh reagents. It has a high thermal stability and shows a high wettability.^25^ The ether oxygen groups of agarose can coordinate with different mobile charge carriers species to provide separators with enhanced ionic conductivities. Additionally, its functional ether (R–O–R) and hydroxyl (−OH) groups facilitate its anchoring onto metallic surfaces, providing a stable electrolyte–electrode interface which increases the battery life span.^26^

The development of a battery separator that exhibits both acceptable electrochemical performance and fast degradability remains a challenging task due to the trade-offs between processability, thermomechanical resistance, ionic conductivity, and electrochemical stability. Accordingly, here we explore the potential of an algae-derived polysaccharide, agarose, in combination with poly(vinyl alcohol) to form thermally stable and water-soluble mesoporous membranes which function as efficient NIB separators.^26^ The cycling stability and electrochemical performance of such separators are studied in symmetric Na/Na and Na_3_V_2_(PO_4_)_3_/Na half-cells. The achieved solubility enables degradable battery separators once a predetermined external trigger (e.g., hot water) is applied, representing a step forward in the development of energy storage devices with lower environmental impact.

## Experimental Section

### Materials

Agarose (low electroendosmosis, CAS: 9012-36-6, negligible sulfate anion amount), glycerol (C_3_H_8_O_3_), poly(vinyl alcohol) (PVA, hydrolysis degree of 99%, *M*_w_ of 85000–124000 g mol^–1^), sodium perchlorate (NaClO_4_), ethylene carbonate (EC), isopropyl alcohol (iPrOH, ≥99.8%,), butanol (BuOH, 99.9%), propylene carbonate (PC), fluoroethylene carbonate (FEC), *N*-methyl-2-pyrrolidone (NMP, 99%), poly(vinylidene fluoride) (PVDF, *M*_w_ of 534000 g mol^–1^), ammonium metavanadate (NH_4_VO_3_, ≥99.5%), citric acid (C_6_H_8_O_7_, ≥99.5%), and ammonium dihydrogen phosphate (NH_4_H_2_PO_4_, 99.999%) have been purchased from Sigma-Aldrich. Polyvinylpyrrolidone (PVP, *M*_w_ of 40000 g mol^–1^) was obtained from Fluka Chemika, Super P carbon black was obtained from TIMCAL Graphite & Carbon, and sodium hydroxide (NaOH, 98.8%) was purchased from VWR Chemicals. All chemicals have been used as received without any further purification. For electrochemical studies, 25 μm thick Celgard membranes (2325) and glass microfiber separators (GF/D, Whatman) were employed as received.

### Fabrication of Mesoporous Agarose Membranes

**Figure 1:**
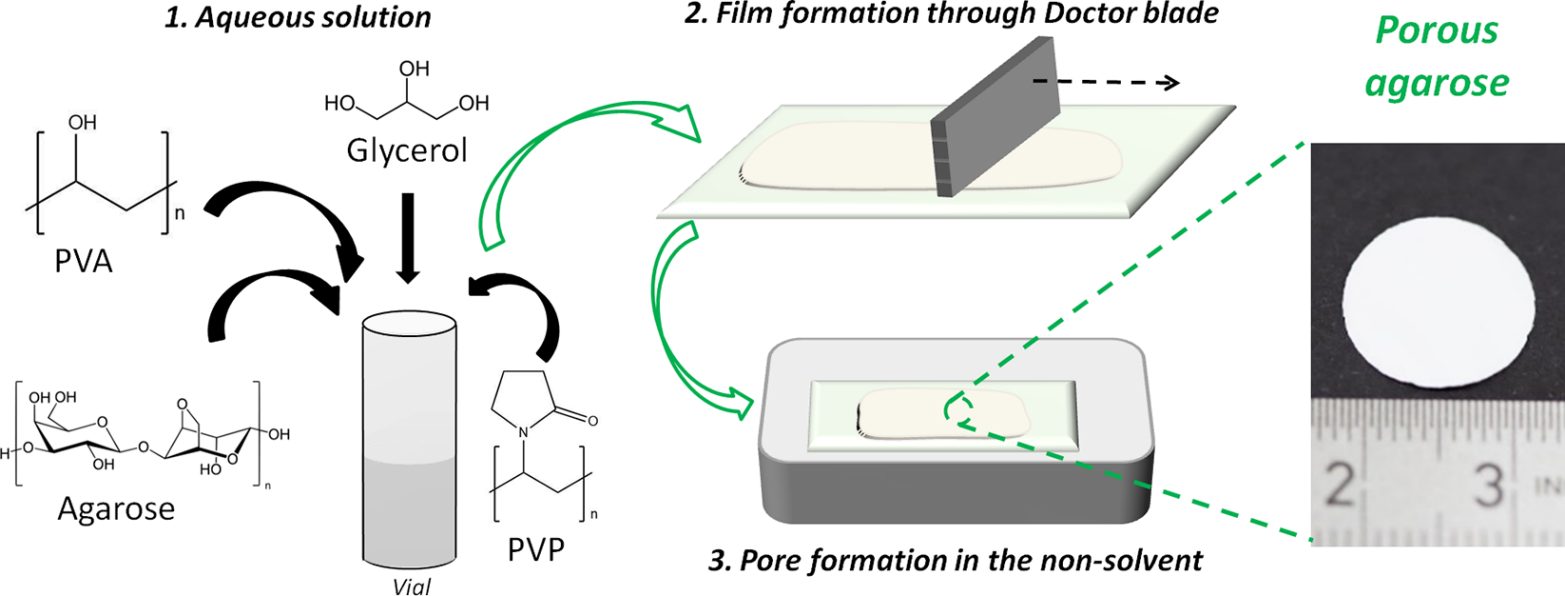
Schematic representation of the fabrication process of agarose-based membranes through NIPS.

As summarized in Figure 1, agarose membranes were synthesized via nonsolvent-induced phase separation (NIPS) by using iPrOH or iPrOH/BuOH (50/50 vol) as nonsolvents.^27^ PVP was used as a pore-forming agent,^28^ PVA provided a phase-separated morphology,^29^ and glycerol was used as a plasticizer. The separators were prepared from a mixture of 1.5% w/v agarose, 1.5% w/v PVA, and ∼10% w/w glycerol (based on agarose + PVA mass) in 25 mL of water, as this ratio provides an adequate balance between solubility and mechanical resistance.^30^ PVP was added in different amounts including 0, 30, and 60 wt % with respect to the total polymer mass (Table S1 provides the exact amount of all the different components along with their nomenclature).

The solution during synthesis was kept between 80 and 90 °C. After a homogeneous solution (magnetically stirred at a speed of 300 rpm for 2 h) was obtained, it was cast onto a clean and flat glass substrate through the doctor blade method with a 1 mm gap to ensure membranes with thicknesses in the range 40–90 μm after the NIPS process. This thickness was selected based on a compromise between cell performance and cell safety. After the doctor blade method, the solution was allowed to cool to room temperature for 30 min. The glass substrate was then introduced into the desired alcohol bath (iPrOH or iPrOH/BuOH) for 180 min. During this process, the water diffused into the alcohol, yielding a thermodynamically stable porous structure.^31^ After this time, the membrane was allowed to dry in an oven at 60 °C for 24 h, and dried films were finally removed from the substrate with the help of a blade.

### Membrane Characterization

Scanning electron microscopy (SEM) analyses were performed by using a DSM 982 Gemini instrument (Zeiss). Prior to the analysis, the samples were sputtered with a 8 nm thick Pt coating. Attenuated total reflectance Fourier transform infrared (ATR-FTIR) spectroscopy results were obtained by using a Bruker Alpha FT-IR spectrometer equipped with diamond ATR optics. A Mettler Toledo TGA/SDTA 851e instrument under an air atmosphere at a heating rate of 10 °C min^–1^ and 50 mL min^–1^ flow was used for thermogravimetric analyses. Differential scanning calorimetry (DSC) traces were recorded with a Mettler Toledo DSC 822e calorimeter under a 50 mL min^–1^ N_2_ atmosphere. Samples having 4 ± 1 mg were sealed in aluminum pans and heated at 10 °C min^–1^ from −30 to 250 °C. Nitrogen sorption experiments were performed on a Quantochrome Autosorb-iQ-C-XR at 77 K with nitrogen (99.999%) and helium (99.999%) provided by PanGas AG, Switzerland. Before each measurement, agarose membranes were degassed in a vacuum at 80 °C for 24 h (specific surface area was determined via the BET method). The mechanical behavior of agarose separators was studied by uniaxial tensile tests using a universal testing machine (Trapezium Shimadzu AGS-X) equipped with a 100 N load cell at a deformation rate of 1 mm min^–1^. Powder X-ray diffraction (XRD) patterns were obtained with a PANalytical Empyrean powder diffractometer in reflection mode using Cu Kα radiation (45 kV, 40 mA). The electrolyte uptake (EU) of agarose membranes was measured after immersion in 1 M NaClO_4_ in EC/PC/FEC (45/45/10 wt %) for 24 h as
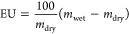
1where *m*_wet_ and *m*_dry_ are the weight of the wet and dry agarose-based separator, respectively. NaClO_4_ was selected as the sodium-containing salt because of its good electrochemical stability up to 5.5 V vs Na, high thermal stability exceeding 550 °C,^32^ an ionic conductivity of 6.35 mS cm^–1^, and widespread application affording comparison with related works.^33^ Moreover, FEC functions as an additive to stabilize the cell performance.^33^

### Ionic Conductivity and Electrochemical Performance

Ionic conductivity and electrochemical performance studies were performed by using a VMP3 Biologic electrochemical workstation. Membranes were always assembled into Swagelok-type cells inside an argon-filled glovebox (H_2_O and O_2_ <0.3 ppm). For ionic conductivity measurements, the membranes were soaked in 1 M NaClO_4_ in EC/PC/FEC (45/45/10 wt %) electrolyte and then sandwiched between two stainless steel rods. The membrane resistance was measured by using a two-probe ac impedance spectroscopy analyzer in the frequency range of 1 mHz–1 MHz with a potentiostatic signal perturbation of 5 mV. The electrochemical stability window was studied by voltammetric measurements, where EC/PC/FEC-soaked (45/45/10 wt %) membranes were sandwiched between a Na disc and a stainless steel rod. The voltammograms were measured in the potential range of 1.8 to −0.3 V, then back to 1.8 V, and up to 5.5 V with a scan rate of 1 mV s^–1^ by using a VMP3 Biologic instrument.

The Na stripping and plating performance was studied under different current densities from ±50 to ±200 μA cm^–2^. To that end, EC/PC/FEC-soaked (45/45/10 wt %) agarose membranes were mounted between two Na-metal discs. Post-mortem SEM micrographs of the separators from symmetric Na/Na cells after 100 h of plating/stripping were obtained as follows. After cycling, the cells were opened inside the glovebox and the separators were pasted onto SEM holders, introduced into a plastic bag, and sealed by vacuum. The samples were then quickly inserted into the SEM instrument for measurements.

NIB testing was performed at room temperature in a half-cell configuration by using sodium vanadium phosphate (Na_3_V_2_(PO_4_)_3_) (NVP) as the cathode. This cathode was selected because of its high Na^+^ mobility and stable 3D host framework,^34^ allowing larger specific capacities than other sodium ion-containing cathodes.^35^ The cathode was obtained based on the approach developed by Feng et al.,^36^ where 80 wt % of Na_3_V_2_(PO_4_)_3_ was mixed with 10 wt % of Super P (conducting additive) and 10 wt % of PVDF binder in NMP. NVP was synthesized by an aqueous sol–gel procedure, followed by annealing.^37^ We dissolved 1.20 g of NaOH, 2.34 g of NH_4_VO_3_, 3.45 g of NH_4_H_2_PO_4_, and 3.84 g of C_6_H_8_O_7_ in 100 mL of deionized water at room temperature using magnetic stirring. Then, the mixture was heated at 80 °C until a dark blue gel was obtained. The gel was transferred into the oven at 60 °C for 48 h to ensure complete drying. The resulting powder was then moved into a tube furnace for calcination at 350 °C for 3 h followed by calcination at 800 °C for 8 h under argon gas. The occurring reaction process can be summarized as
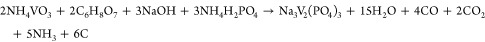
2Cells were assembled with a Na foil of 11 mm diameter as anode and agarose membranes soaked in 1 M NaClO_4_ in EC/PC/FEC (45/45/10 wt %) electrolyte as separators. Galvanostatic charge–discharge cycling was performed in the 2.8–3.8 V (vs Na/Na^+^) range at different rates from C/10 to 1C (1C = 117.6 mA g^–1^).^36^ For the sake of comparison, the performance of Na_3_V_2_(PO_4_)_3_/Na half-cells containing Celgard 2325 soaked in 1 M NaClO_4_ in EC/PC/FEC (45/45/10 wt %) was also investigated.

## Results and Discussion

### Morphological Characterization

The focus of our work is on the fabrication of separators based on agarose that offer a rapid and stable Na^+^ transport between electrodes.^26,37^ As obtaining highly porous membranes is a prime requisite toward efficient battery separators,^16^ our efforts were directed toward the optimization of porous structure using nonsolvent-induced phase separation (NIPS). Preliminary experiments indicated that the agarose concentration for the dissolution process should be in the range 1.5–3 wt % because lower concentrations result in the loss of the film-forming ability (failing to form free-standing membranes), while higher concentrations yield large viscosities that are difficult to be processed through the doctor blade method. We also found that upon glycerol addition the mechanical ductility of the final membranes can be improved,^38^ which could a priori help to obtain improved interfacial contact with the metallic Na, providing batteries with lower interfacial resistances.^17^

**Figure 2:**
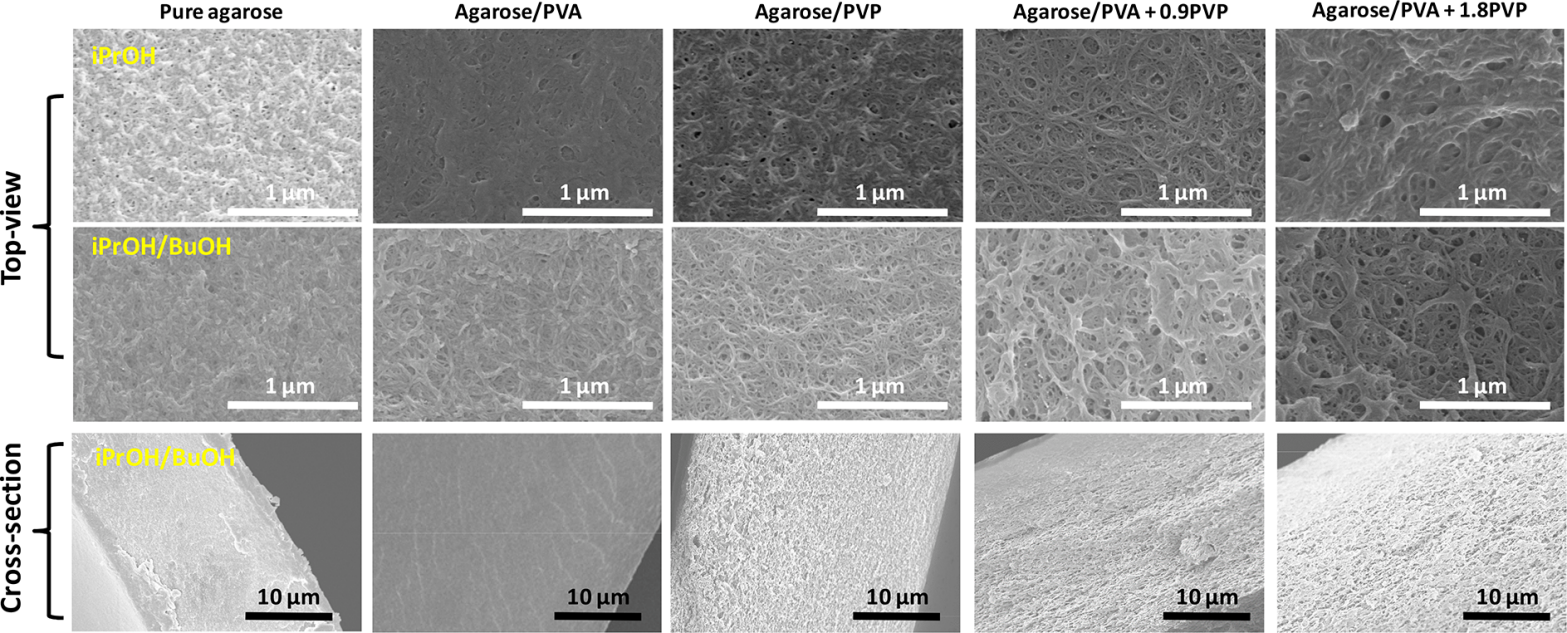
Representative SEM micrographs showing the top view of fabricated membranes obtained upon immersion into iPrOH (upper row) and iPrOH/BuOH (middle row). Cross-sectional SEM images corresponding to membranes obtained with iPrOH/BuOH are shown in the bottom row.

Upon immersion of the wet films into the nonsolvent, the demixing process yields a thermodynamically stable porous structure.^31^ We explore two different alcohols as nonsolvents, iPrOH and its 50/50 vol mixture with BuOH, to create the pores. Figure 2 shows the top-view and cross-sectional SEM images of the agarose membranes. Pure agarose fails to form a porous membrane after immersion in either iPrOH or iPrOH/BuOH (Figure 2, first column). Therefore, we incorporated PVA as a representative water-soluble polymer with good pore-forming ability into agarose solutions to enhance the porosity of the membranes.^39^ We found that after NIPS in iPrOH a relatively dense film with few pores was achieved, while iPrOH/BuOH resulted in a membrane with porous features (Figure 2, second column). Unfortunately, its corresponding cross-sectional SEM image proves that this approach fails to form a three-dimensional porous structure across the entire membrane thickness. As larger porosities are preferred to enable a homogeneous and stable ion electrodeposition onto electrodes, PVP was also added to facilitate the pore formation process as it is a preferred additive for the fabrication of porous membranes thanks to its water solubility and miscibility with many polymers.^28^ It can be observed that the agarose membrane comprising PVP forms a porous structure (Figure 2, third column). By combining PVA and PVP (fourth and fifth columns in Figure 2, see Table S1 for the composition), we can obtain membranes with a highly porous character, which have pores of tens of nanometers and which are interconnected from the surface through the whole membrane thickness. Such morphology is especially interesting to function as a battery separator as it facilitates high electrolyte uptake and homogeneous ion transport across the separator.^40^ As shown in Figures S1 and S2, this is translated into optically white samples characteristic of porous membranes. In contrast, solid films with little porosity obtained without PVA are transparent. Based on the apparent density of the membranes, their porosities can be calculated according to eqs S1 and S2. As summarized in Table S2, porosities in the range 52–76% were obtained for the films containing agarose, PVA, and PVP, notably higher than the porosities of 35–55% characteristic of commercial polyolefin battery separators.^41^

Besides the electron microscopy studies in Figure 2, membrane porosity was further evaluated through N_2_ adsorption–desorption experiments. As shown in Figure S3a, agarose-based membranes present a maximum Brunauer–Emmett–Teller (BET) surface area (*S*_BET_) of 66.6 m^2^ g^–1^. As evidenced by the *S*_BET_ decrease to 24.4 m^2^ g^–1^, the incorporation of PVP, when iPrOH is used solely as the nonsolvent, is detrimental for the porosity. In contrast, the addition of BuOH into the nonsolvent enhances the pore formation in the membrane. Such improvements may arise from the different boiling points of iPrOH and BuOH (83 vs 118 °C, respectively) which slows down the solvent exchange kinetics. Furthermore, the nonpolar nature of BuOH slows down the diffusion process, increasing the time for the demixing process.^42^ In any case, the obtained surface areas are higher than the *S*_BET_ of 10.8 m^2^ g^–1^ measured for the commercial glass fiber separator.^27^ The type IV isotherm with H_2_ hysteresis provides evidence for the mesoporous morphology, which is in line with the cumulative surface area plot shown in Figure S3b, confirming that pores in the range 5–25 nm contribute primarily to the surface area. These mesopores are beneficial for battery separators as they enable a uniform ion flux across the electrodes, resulting in a stable ion deposition/stripping which is critical to avoid dendrite formation.^43^

### Membrane Physicochemical Characterization

**Figure 3:**
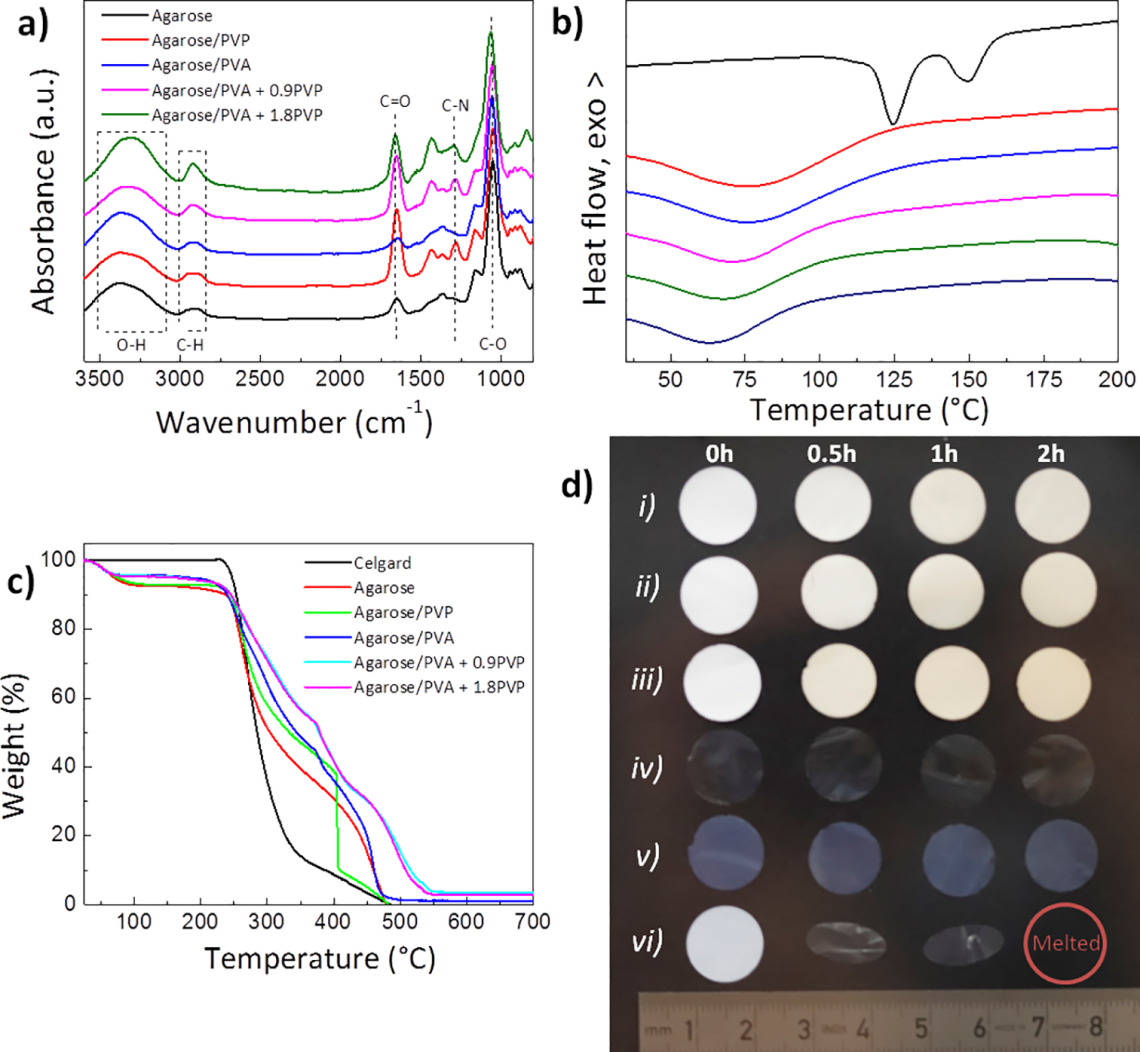
(a) ATR-FTIR spectra; (b) DSC curves; (c) TGA traces under air atmosphere; and (d) digital photographs showing the thermal stability of the agarose-based membranes and Celgard obtained in iPrOH/BuOH at 160 °C for different times (0, 0.5, 1, and 2 h): (i) agarose/PVA + 1.8 PVP; (ii) agarose/PVA + 0.9 PVP; (iii) agarose/PVA; (iv) agarose/PVP; (v) pure agarose; and (vi) Celgard.

Attenuated total reflectance Fourier transform infrared (ATR-FTIR) spectra in Figure 3a show the characteristic bands of agarose and PVA (in iPrOH/BuOH), where the broad absorption band between 3600 and 3000 cm^–1^ corresponds to the O–H stretching vibration of the alcohol groups in agarose and PVA and to the as-formed intermolecular hydrogen bonds between both blended components.^44^ The region between 3000 and 2800 cm^–1^ shows a superposition of the C–H bond stretching of the alkane group in agarose and C–H stretch from alkyl groups in PVA,^24^ while the sharp band at 1072 cm^–1^ arises from the deformation mode of the C–O groups in agarose.^25^ The presence of PVP is observed by the bands at 1660 cm^–1^ (C=O) and 1290 cm^–1^ (C–N).^45^

The thermal stability of the membrane, a prime requisite toward improved safety, has been assessed by differential scanning calorimetry (DSC) and thermogravimetric analysis (TGA). As denoted by the DSC curves in Figure 3b, the membranes present a broad endothermic peak in the 40–110 °C temperature range arising from dehydration of agarose.^25^ In comparison with Celgard, which displays two sharp melting peaks at 125 and 150 °C originating from polyethylene and polypropylene, respectively,^16^ no signs of melting or thermal degradation are observed in the temperature range studied. TGA curves under air atmosphere in Figure 3c show an initial smooth loss of 5–7 wt % due to the adsorbed moisture evaporation. Upon further heating, a pronounced mass loss occurs in the range of 250–460 °C, which is ascribed to agarose pyrolysis.^25^ Although all samples present a similar onset of thermal degradation at 242–250 °C, agarose-based membranes show enhanced thermal stability in comparison with Celgard. Membranes containing PVP present improved stability, reaching a 50% weight loss at 379 °C in comparison with the 284 °C observed for Celgard.

This resistance against thermally induced degradation of agarose is beneficial for battery separators with reduced likelihood of thermal runaway. To confirm the improved resistance at high temperatures, the thermally induced shrinkage after treating all the separators at 160 °C for different times is shown in Figure 3d. In comparison with the commercial polyolefin separators, which shrink above 130 °C,^16^ agarose-based membranes display a good thermal dimensional stability with no signs of macroscopic shrinkage or expansion. This improved thermal stability was also translated at the nano/micro scale, where agarose-based membranes kept their morphology intact after being heat treated at 160 °C for 2 h (Figure S4). Overall, these results indicate the potential of agarose to electrically isolate anode and cathode even at high temperatures.

Tensile properties, including Young’s modulus (*E*), tensile stress at break (σ_b_), and elongation at break (ε_b_), determine the suitability of agarose membranes as battery separators by providing information about the ability to withstand mechanical stresses associated with rough surface electrodes and dendrite growth.^46^ The mechanical properties of the membranes were evaluated by uniaxial tensile tests and the representative stress–strain curves are summarized in Figure S5 (see Table S3 for main representative tensile test parameters). Agarose membranes display a semiductile behavior with *E* values in the range of 73–219 MPa and ε_b_ ranging from 10.1 to 33.9%. Membranes immersed in iPrOH/BuOH show a decreased modulus and lower strain at break due to the increased porosity of the membrane. However, the obtained combination of *E* and a semiductile character (in comparison with the poor ductility of Celgard 2400; ε_b_: 3%)^47^ allows easy handling of the membranes during battery assembly while providing good resistance to dendrite puncture.

**Figure 4:**
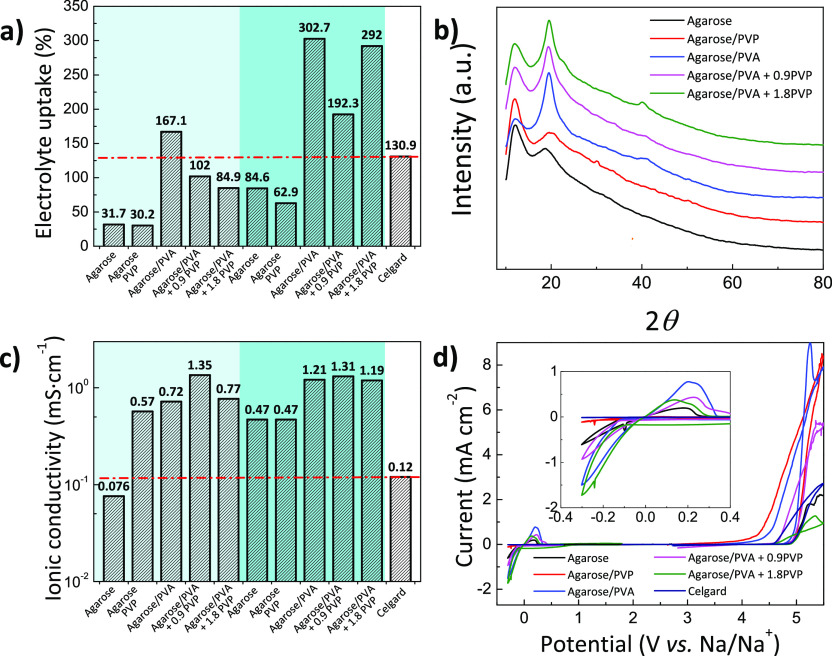
(a) Electrolyte uptake; (b) XRD patterns (iPrOH/BuOH); (c) Na^+^ conductivity; and (d) electrochemical stability window of membranes (iPrOH/BuOH). Dark cyan regions in (a) and (c) correspond to membranes obtained upon immersion into a 50/50 iPrOH/BuOH bath. The inset in (d) magnifies the −0.4 to 0.4 vs Na/Na^+^ voltage window.

To confirm whether or not the interconnected mesoporous network with a large specific surface area translate into an efficient electrolyte soaking, the electrolyte uptake (EU) of the different membranes was measured according to eq 1. As summarized in Figure 4a, neat agarose and agarose/PVP membranes present a poor EU (<85 wt %). Upon PVA incorporation, EU values of 167.1 and 302.7 wt % are achieved when immersed in iPrOH and iPrOH/BuOH, respectively. This electrolyte uptake, higher than the 130.9 wt % shown by commercial Celgard, is ascribed to the abundant oxygen-containing functional groups of agarose, providing a better affinity, and to the decreased surface tension and capillary action due to the porosity.^48^ The measured electrolyte uptake is also considerably larger than the 131 wt % observed for cellulose-derived separators^27^ or the 151 wt % of a nonwoven poly(vinylidene fluoride–hexafluoropropylene),^49^ indicating that agarose membranes could provide an efficient medium for Na^+^ transference.

As a predominantly amorphous structure is commonly related to favorable ion diffusivity,^50^ X-ray diffraction experiments of the membranes obtained after immersion in iPrOH/BuOH bath have been performed to study the crystalline structure of the membranes. All the diffractograms in Figure 4b present two main broad peaks located at 2θ = 11.2° and 19.8°, corresponding to the overlapping diffraction peaks of the ordered stacking of agarose polysaccharide rings,^51^ and the diffraction peak of PVA at 2θ = 19.5°, corresponding to its (101) crystal plane.^52^ The absence of sharp diffraction peaks and the presence of a broad amorphous halo indicate the coexistence of large amorphous regions with few crystalline phases. A crystallinity degree of only 10–20% is obtained for the membranes thanks to the predominantly amorphous character of agarose.^53^ These crystallinity values remain well below the 95% observed for *Cladophora* cellulose separators^54^ or the 57% for PVDF separators,^55^ providing a reduced barrier for ion migration because ions can freely move within the large amorphous regions.^56^

The high porosity in combination with the predominantly amorphous character and the high electrolyte uptake suggest that these membranes should have favorable ionic conductivities. The ionic conductivity and electrochemical performance were evaluated by using a 1 M NaClO_4_ in EC/PC/FEC (45/45/10 wt %) electrolyte. In spite of the fire and explosive hazard of NaClO_4_ (GHS Hazard Statements: H271, H302, H319, and H373), this salt is a popular choice for NIBs due to its good solubility in different solvent(s), high melting point of 468 °C, and high ionic conductivity of 6.4 mS cm^–1^.^57^ Its use allows a cross-comparison with reported NIB systems having a Na_3_V_2_(PO_4_)_3_ cathode, providing good evidence for the viability of agarose-based separators.^27,58,59^ Based on the Nyquist impedance plots in Figure S6, Na^+^ conductivity (σ_*i*_, S cm^–1^) values of electrolyte-soaked membranes are calculated as follows:
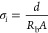
3where *d* accounts for the separator thickness, *R*_b_ is the bulk resistance determined from the intercept of the curve with the real impedance axis, and *A* is the contact area of the separator with the stainless steel electrode. For all the samples the Nyquist plots show straight lines with no semicircles, indicating the ionically conducting nature of the membranes. With a maximum ionic conductivity of 1.35 × 10^–3^ S cm^–1^ (Figure 4c), the Na^+^ conductivity of our membranes is above the 0.12 × 10^–3^ S cm^–1^ shown by commercial Celgard. These conductivities are also superior to the reported values of 1.02 × 10^–3^ S cm^–1^ for cellulose acetate^60^ or the 0.066 × 10^–3^ S cm^–1^ for chitin nanofiber membranes.^21^ Possible reasons are ascribed to the coordination bonds for the Na^+^ as mobile charge species provided by the ether oxygen groups of agarose and hydroxyl groups of PVA.^61^

**Figure 5:**
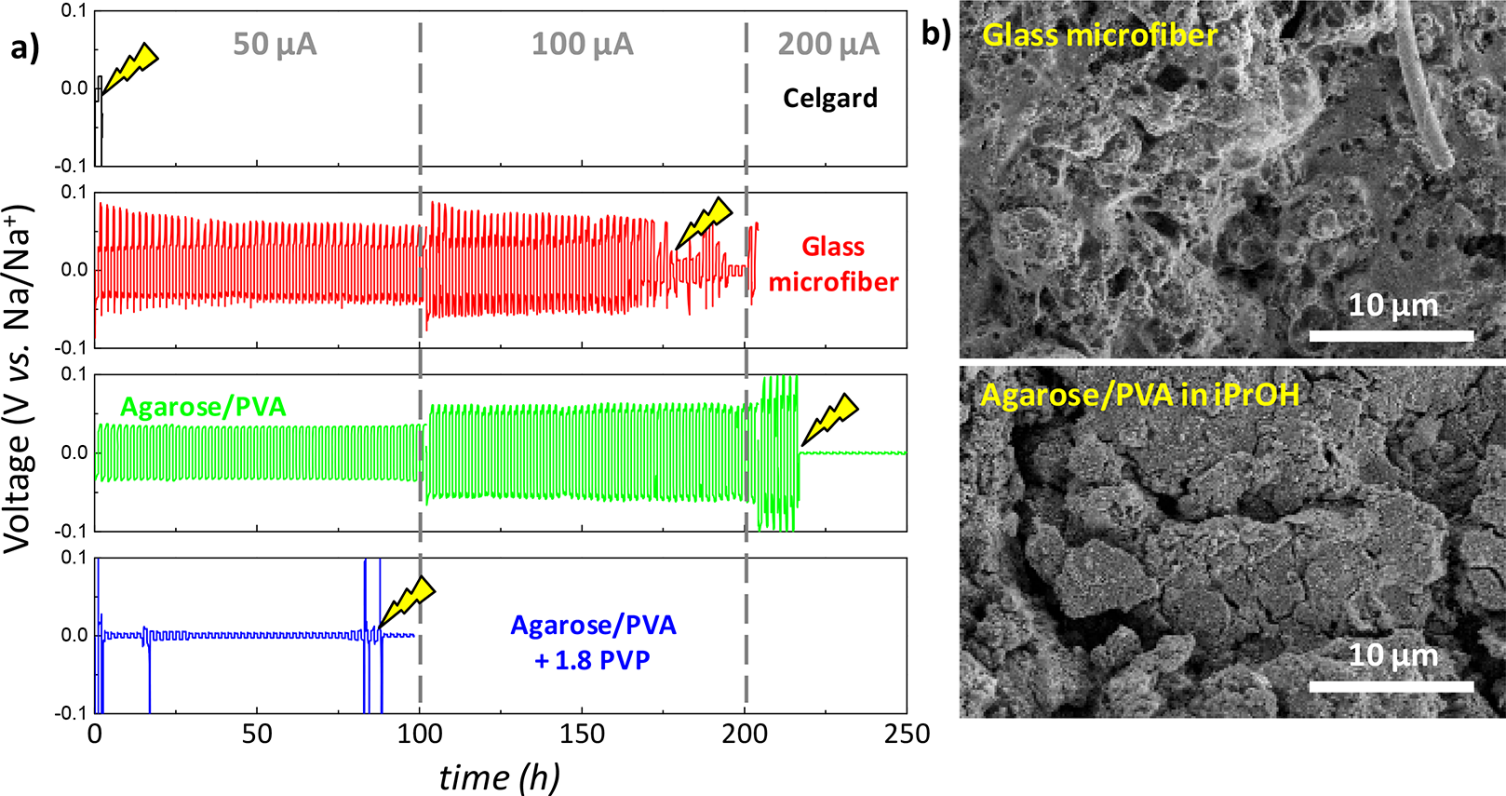
(a) Room temperature voltage vs time curves for a symmetric Na/Na cell for Na plating/stripping at current densities from ±50 to 200 μA cm^–2^ for commercial Celgard and glass microfiber separators together with agarose membranes. The lightning signals the occurrence of short circuit. (b) SEM micrographs of Na metal surfaces after galvanostatic cycling of symmetric Na cells with a glass microfiber separator and agarose/PVA separator prepared in iPrOH.

Considering morphology, thermal/dimensional stability, amorphous character, and ionic conductivity, the agarose membranes are attractive candidates for battery separators. However, the electrochemical stability also plays a critical role in determining whether or not agarose membranes can be applied for such a purpose.^17^ Accordingly, Figure 4d shows a combined cyclic voltammetry and linear sweep voltammogram curves in the −0.3 to 5.5 V range (cyclic voltammetry from −0.3 to 1.8 V and linear sweep voltammetry from 1.8 to 5.5 V are combined and measured using a stainless-steel working electrode and metallic Na counter electrodes at 1 mV s^–1^). The membranes are stable up to 4.5 V vs Na/Na^+^, which makes them also useful for other high-voltage NIB cathodes.^62^ As shown in the inset of Figure 4d, a neat agarose membrane presents reversible sodium deposition and dissolution currents with an anodic peak of 0.20 mA cm^–2^ located at ∼0.19 V vs Na/Na^+^.^63^ The intensity of the peak is increased to 0.76 mA cm^–2^ when PVA is incorporated during the NIPS process (in iPrOH/BuOH) due to an increased resistance to Na dissolution/deposition process (also confirmed by Na plating/stripping experiments in Figure 5). Importantly, the presence of PVP lowers the maximum intensity of the anodic peak to 0.37 mA cm^–2^, suggesting a more homogeneous Na plating/stripping onto stainless steel electrodes with efficient anodic Na dissolution and cathodic deposition at the Na/separator interface.

### Electrochemical Performance

The electrochemical performance of agarose membranes has been assessed in a symmetric Na/Na cell configuration and in Na_3_V_2_(PO_4_)_3_/Na half-cells. Based on the morphological and physicochemical results presented above, agarose/PVA membranes prepared in both iPrOH and iPrOH/BuOH have been selected as the most promising materials as NIB separators. The long-term galvanostatic cycling in a symmetric Na/Na cell at room temperature enables studying the reversibility of the Na^+^ transport through the membranes (Figure 5),^27^ where the negative and positive potentials represent Na-metal stripping and plating, respectively. Agarose-based membranes show an overpotential of 36, 5, and 2 mV at a current density of 50 μA cm^–2^ (agarose/PVA, agarose/PVA + 1.8 PVP, and agarose/PVA iPrOH/BuOH, respectively), which is lower than the overpotential of 83 mV shown by the glass microfiber. Such reduced overpotential arises from the good interfacial compatibility and intimate contact of agarose membranes with the Na surfaces due to the hydroxyl and ether groups that facilitate an intimate contact with metallic surfaces.^37^ Besides, agarose-based membranes present a nearly square wave shape compared to the voltage fluctuations shown by the glass microfiber separator (see Figure S7 for a magnified view of the voltage vs time curves in the 90–100 h range). Such deviation from the square-wave shape indicates a continuous growth of a resistive solid electrolyte interphase onto the surface of the Na metal.^64^

Figure 5b shows the morphology of the surfaces of the Na metal after galvanostatic cycling of symmetric Na cells by using a glass microfiber separator, and agarose/PVA separator prepared in iPrOH. The glass microfiber results in a rough surface with significant microstructural irregularities, while a more homogeneous surface is observed when an agarose membrane is used as a separator. It can be observed that the Na surface becomes more regular for the agarose-based membrane, which is in line with the lower overpotential observed in Figure 5a. Additionally, post-mortem SEM and energy-dispersive X-ray spectroscopy analysis of separators (after 100 h of plating/stripping) in Figure S8 reveal the presence of larger Na deposits for the glass microfiber (when comparing with agarose-based separator). A stable and homogeneous Na deposition is usually manifested in enhanced battery life spans.^65^ Indeed, those morphological results are in line with the life-cycle stability of membranes (Figure 5a,b), where agarose-based separators withstand up to 215 h at current densities up to 200 μA cm^–2^. In comparison, Celgard and glass microfiber separators show an early short-circuit after only 2.1 and 180 h (at 50 and 100 μA cm^–2^), respectively. The long-term stability and good Na^+^ insertion/extraction reversibility provided by the agarose-based membrane are ascribed to its mesoporous morphology and highly ionically conducting character, enabling efficient Na^+^ electrodeposition onto electrodes while delaying Na dendrite formation.^66^ Overall, those results highlight the potential of algae-derived polysaccharides to obtain long life span battery separators which efficiently transport Na^+^ and mitigate dendrite formation.

**Figure 6:**
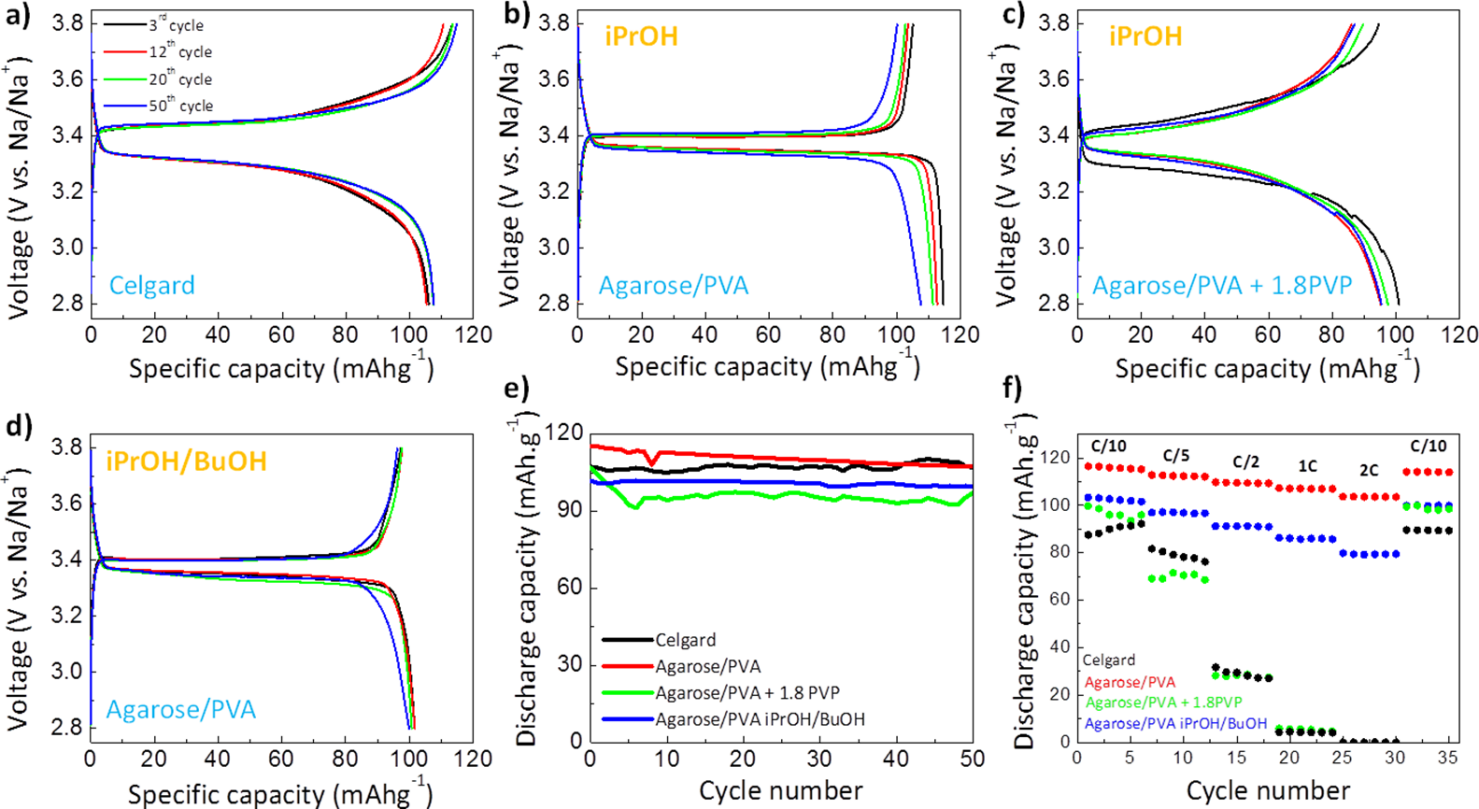
(a–d) Galvanostatic charge/discharge profiles at C/10 for agarose membranes together with (e) their corresponding evolution of the discharge capacity. Galvanostatic charge–discharge profiles for Celgard separator are provided for comparison. (f) Discharge capacities of agarose membranes at different C-rates. A rate of 1C is equivalent to a current density of 117.6 mA g^–1^.

Agarose-based membranes were assembled into Na_3_V_2_(PO_4_)_3_/Na half-cells to evaluate their potential as NIB separators. Given its safety and relatively good performance, Na_3_V_2_(PO_4_)_3_ as a cathode is a common choice when studying NIB performance, enabling a comparison between separators of different compositions.^67^ For cathode manufacturing, 10 wt % of carbonaceous fillers (Super-P) is incorporated into the Na_3_V_2_(PO_4_)_3_ to enhance its electrical conductivity, while 10 wt % of PVDF is added as a binder to protect the cathode against pulverization during (de)insertion. Figure 6a–d summarizes the galvanostatic charge–discharge curves in the 2.8–3.8 V (vs Na/Na^+^) window at C/10 (1C = 117.6 mA g^–1^) obtained for agarose-based separators.^36^ For an easier understanding, charge/discharge curves of Celgard (as a representative commercial separator) are also shown. Generally, agarose-containing membranes deliver increased capacities not only during the first cycle but also in the successive charge/discharge processes. Initial discharge capacities of 107, 116, 106, and 102 mAh g^–1^ are observed for Celgard, agarose/PVA (iPrOH), agarose/PVA + 1.8PVP (iPrOH), and agarose/PVA (iPrOH/BuOH) separators, respectively. Our membranes surpass the specific capacities delivered by other separators in Na_3_V_2_(PO_4_)_3_/Na half-cells, such as those based on carboxymethyl cellulose (94 mAh g^–1^ at C/10),^27^ polysulfonamide-based separator (101 mAh g^–1^ at C/5),^68^ polypropylene separator (91 mAh g^–1^ at C/5),^68^ or chitin nanofiber separator (∼70 mAh g^–1^ at C/10) (see the comparison of the electrochemical performance of different separators in a Na_3_V_2_(PO_4_)_3_/Na configuration in Table S4).^21^

Galvanostatic charge/discharge curves of Na_3_V_2_(PO_4_)_3_/Na half-cells present a plateau at nearly 3.4 V vs Na/Na^+^ as a result of the two-phase transition of the V^3+^/V^4+^ redox couple (Na_3_V_2_(PO_4_)_3_ ↔ NaV_2_(PO_4_)_3_).^67^ It should be noticed that the charge/discharge curves of batteries containing agarose membranes are characterized by a very flat voltage plateau with low electrochemical polarization of 42 mV (the difference between charge and discharge plateaus), in comparison with a polarization of 162 mV for Celgard. This reduced polarization indicates an enhanced ionic diffusion between the cathode and anode enabled by the agarose-containing membrane. Importantly, as shown in Figure 6e, cells having agarose-based separators retain >93% of their initial capacity after 50 cycles at C/10. A higher Coulombic efficiency (CE) is observed for the agarose-based separators in comparison with that obtained for Celgard (see the CE plot in Figure S9). CE values exceeding 100% for agarose-based separators can arise from the electrons generated during irreversible electrochemical reactions which are acquired by the current collector and subsequently counted into the CE calculation.^69^ Moreover, a larger amount in electrolyte content (due to notably higher electrolyte uptake of agarose membranes) can deliver larger CE values.^69^ Importantly, Figure 6f demonstrates the good rate capability of agarose membranes, especially at high C-rates, where the initial C/10 capacity is recovered after 30 cycles at different C-rates (from C/10 to 2C). The mesoporous morphology of agarose-based membranes, their amorphous/hydrophilic character, and large electrolyte uptake, enabling large ionic conductivities, stable interfacial contact with Na anode, and uniform Na^+^-ion flux between electrodes, provide a means to reduce the Ohmic polarization during cycling, enhancing the capacities, CE and rate performance.

## Conclusions

The design of battery separators from a circular economy perspective depends on balancing the often conflicting requirements such as renewability, acceptable electrochemical performance, and degradability. Here we attain a fine balance of these stringent requirements by using water-soluble and nontoxic agarose, a biopolymer of marine origin. Free-standing membranes with interconnected mesopores are obtained upon optimization of the nonsolvent-induced phase separation process, enabling uniform Na^+^ flux between electrodes. The hydroxyl and ether groups of agarose enable an intimate interfacial contact with the Na metal surface, providing homogeneous sodium plating/stripping with efficient Na^+^ electrodeposition capable of operating at current densities as high as 200 μA cm^–2^. When evaluated in Na_3_V_2_(PO_4_)_3_/Na half-cells, agarose-based separators deliver 108 mAh g^–1^ after 50 cycles, with a remarkable rate capability. Overall, our membranes surpass commercially available separators in terms of thermal stability, electrolyte uptake, ionic conductivity, and Na plating/stripping stability. The results provide cues for the development of water-degradable NIBs comprising renewable materials, which are expected to play a relevant role in the future sustainable energy storage landscape.

## Supporting Information Available

Optical photographs, N_2_ adsorption–desorption isotherms, SEM micrographs, tensile stress–strain curves, Nyquist plots, current–time profiles, post-mortem SEM analysis of the membranes and Coulombic efficiency of agarose membranes; tables showing sample composition, porosity estimation, main representative tensile test parameters, and comparison of the electrochemical performance of different separators in a Na_3_V_2_(PO_4_)_3_/Na half-cell (PDF)
