# Supplementary material for: Stable Na Electrodeposition Enabled by Agarose-Based Water-Soluble Sodium Ion Battery Separators
Source: ACS Appl Mater Interfaces. 2021 Apr 29;13(18):21250–60. doi: 10.1021/acsami.1c02135 (PMC9161220; doi:10.1021/acsami.1c02135)
Supplement: Supplementary file 1 — am1c02135_si_001.pdf [file am1c02135_si_001.pdf]

Supporting Information

Stable Na Electrodeposition Enabled by Agarose-Based Water-Soluble Sodium Ion Battery Separators

Alazne Ojanguren,[†#] *Neeru Mittal,*[†#] *Erlantz Lizundia*[‡,§*] *and Markus Niederberger*[†*]

[†] Laboratory for Multifunctional Materials, Department of Materials, ETH Zürich, Vladimir-Prelog-Weg 5, 8093 Zurich, Switzerland.

[‡] Life Cycle Thinking Group, Department of Graphic Design and Engineering Projects, Faculty of Engineering in Bilbao, University of the Basque Country (UPV/EHU), Bilbao 48013, Spain.

[§] BCMaterials, Basque Center for Materials, Applications and Nanostructures, UPV/EHU Science Park, 48940 Leioa, Spain.

#: Equal contribution

*: Corresponding authors: erlantz.liizundia@ehu.eus; markus.niederberger@mat.ethz.ch

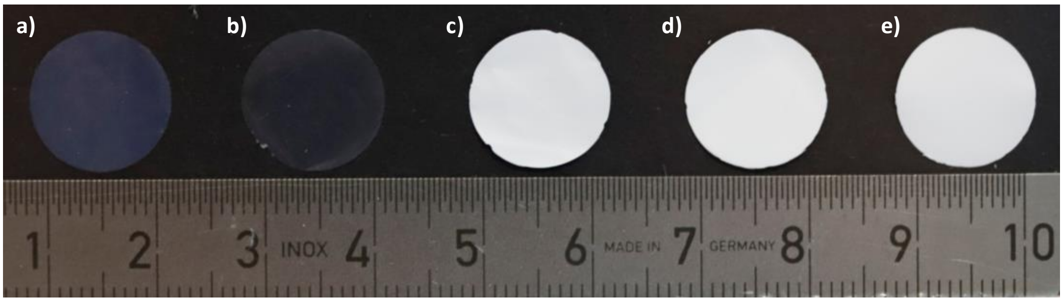

Figure S1. Digital photographs of the membranes with diameters of 13 mm after immersion into an iPrOH bath for 3 h and subsequent drying in the oven at 60 °C: (a) pure agarose; (b) agarose/PVP; (c) agarose/PVA; (d) agarose/PVA + 0.9PVP; (e) agarose/PVA + 1.8PVP.

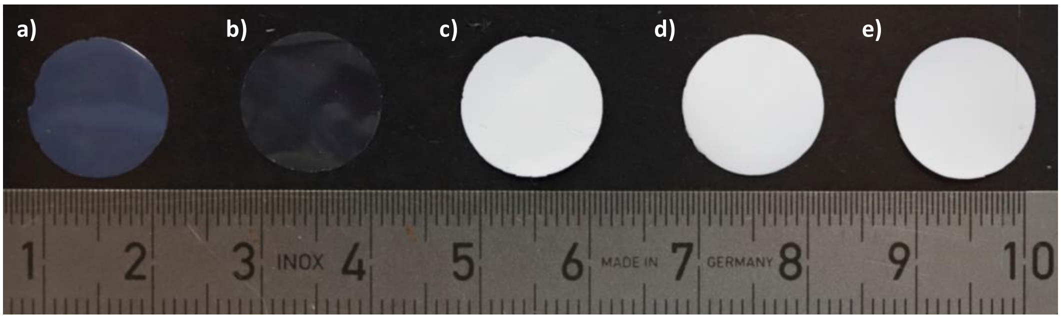

Figure S2. Digital photographs of the membranes with diameters of 13 mm after immersion into a 50/50 iPrOH/BuOH bath for 3 h and subsequent drying in the oven at 60 °C: (a) pure agarose; (b) agarose/PVP; (c) agarose/PVA; (d) agarose/PVA + 0.9PVP; (e) agarose/PVA + 1.8PVP.

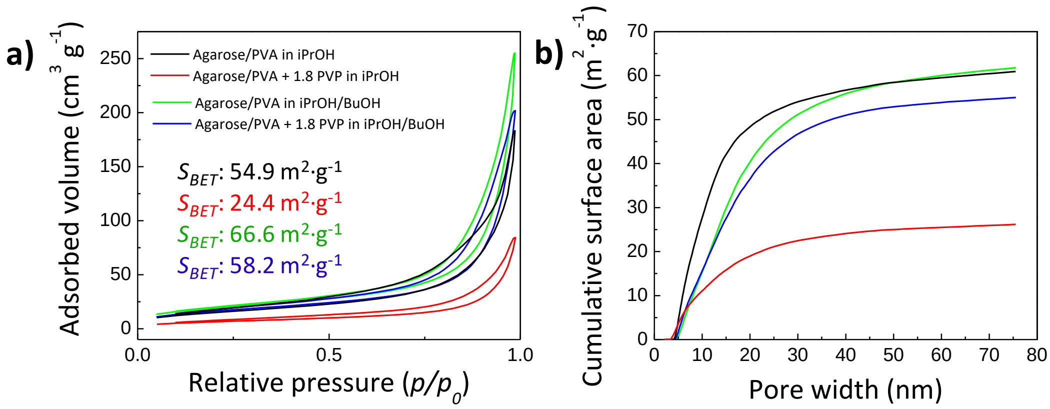

Figure S3. (a) N_2 adsorption–desorption isotherms of the different membranes and (b) the corresponding DFT pore width analysis on the desorption branch of the BET isotherm. Inset (a): Calculated Brunauer–Emmett–Teller (BET) specific surface area (S_{BET}).

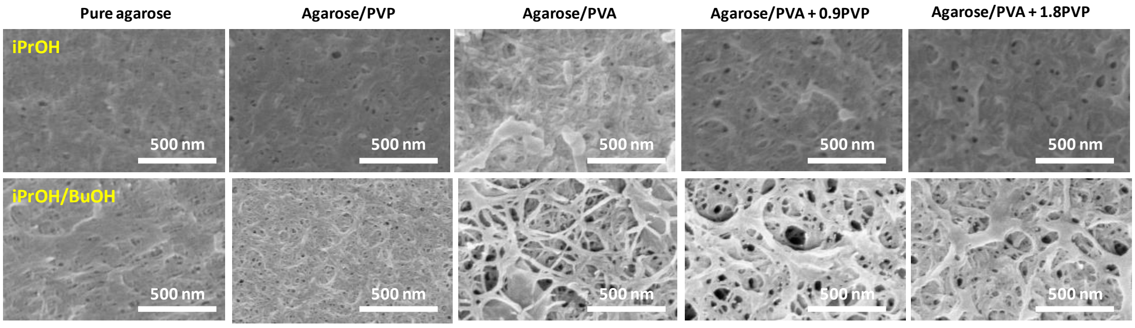

Figure S4. Representative SEM micrographs of the different membranes after heating at 160 ºC for 2 hours.

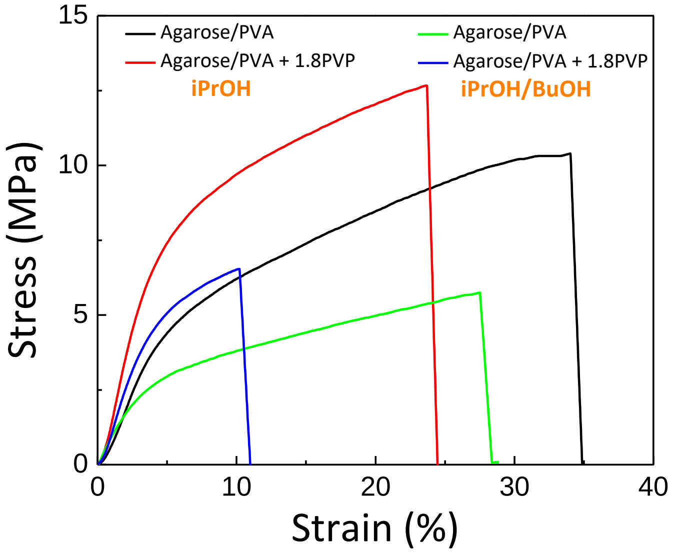

Figure S5. Representative stress–strain curves for different agarose membranes.

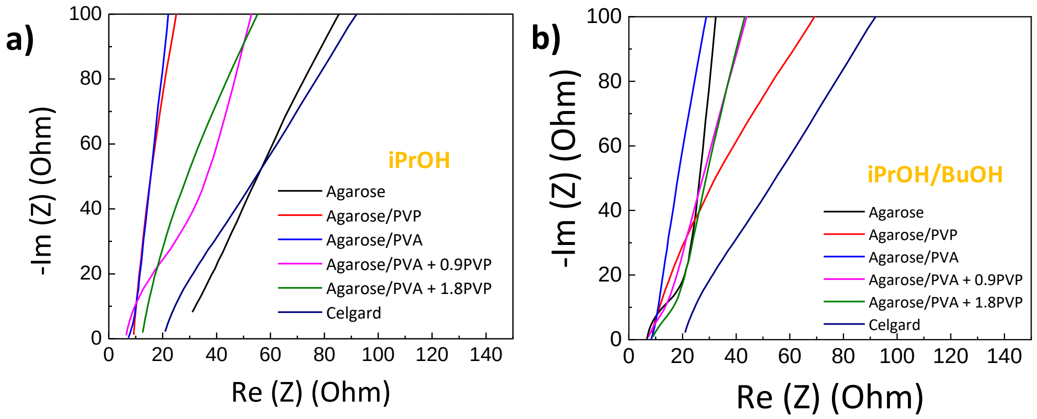

Figure S6. Nyquist impedance plot of the different membranes: (a) after immersion in iPrOH and (b) iPrOH /BuOH (frequency range from 1 mHz to 1 MHz with a potentiostatic signal perturbation of 5 mV).

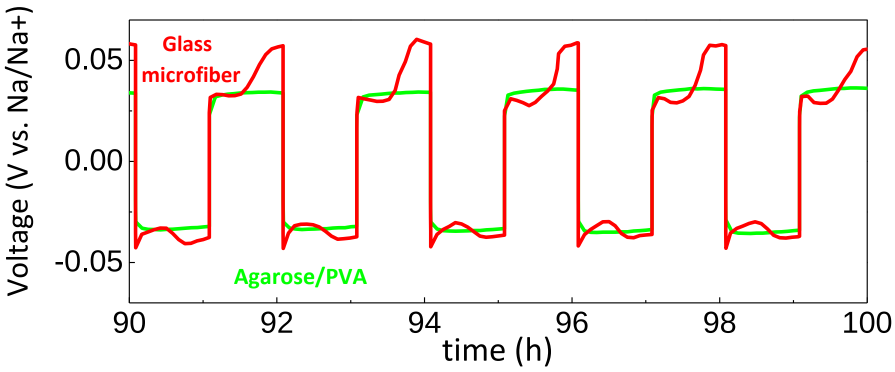

Figure S7. Magnified view of the room-temperature voltage *vs.* time curves for a symmetric Na/Na cell tested for Na plating/stripping at a current density of ± 50 μA·cm^{-2} in the 90 to 100 h range.

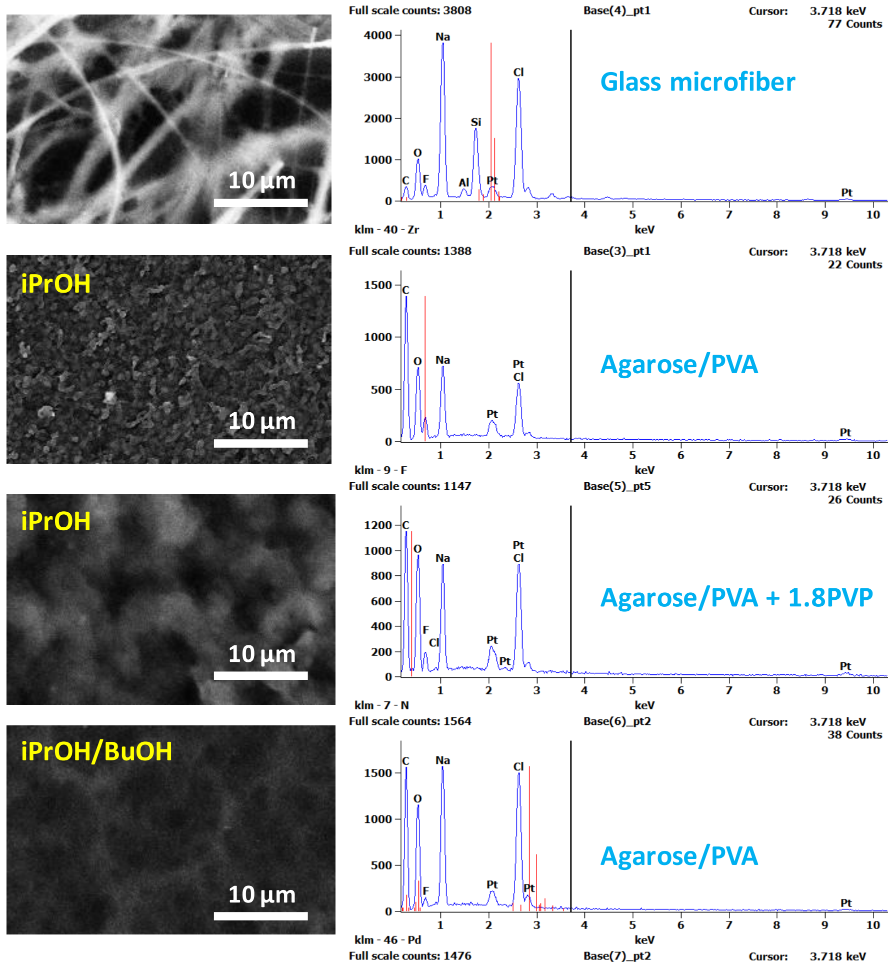

Figure S8. Post-mortem analysis of the separator after 100 h of plating/striping: SEM micrographs showing the surface of the separators after symmetric cycling between two metallic Na electrodes together with their corresponding energy-dispersive X-ray spectroscopy (EDX) analysis. The glass microfiber separator shows a pronounced Na peak (Ka = 1.04 keV). The presence of Na is due to the deposition of the sodium from the $NaClO_4$ in the liquid electrolyte and the Na from the electrodes.

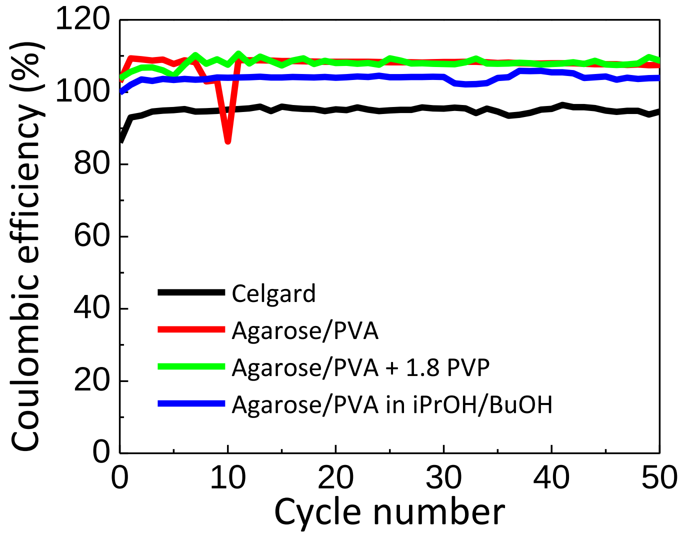

Figure S9. Coulombic efficiency at C/10 for agarose membranes.

Table S1. Sample codes together with the amounts of agarose, PVA, PVP and glycerol used for their synthesis.

Sample code	Agarose (g)	PVA (g)	PVP (g)	Glycerol (g)
Agarose	0.750	0	0	0.083
Agarose/PVP	0.750	0	0.450	0.083
Agarose/PVA	0.375	0.375	0	0.083
Agarose/PVA +0.9PVP	0.375	0.375	0.225	0.083
Agarose/PVA +1.8PVP	0.375	0.375	0.450	0.083

The porosity of the membranes was calculated according to Eqs S1 and S2 measuring the apparent density of the samples.

$$Porosity\ (\%) = \left(1 - \frac{\rho_0}{\rho_c}\right) \times 100 \qquad \text{(S1)}$$

$$\rho_0 = \frac{mass}{apparent\ volume} \qquad \text{(S2)}$$

where ρ_0 accounts for the apparent density and ρ_c represents the bulk density of agarose and PVA (1.00 $g{\cdot}cm^{-3}$ for agarose and 1.19 $g{\cdot}cm^{-3}$ for PVA). The porosity of commercial polyolefin separators is in the range of 35-55 %.

Table S2. Porosity estimation according to Eqs S1 and S2. First five rows correspond to samples immersed in iPrOH, while the rest corresponds to those in iPrOH/BuOH.

Sample	Mass (g)	Apparent volume (cm^3)	ρ_c ($g.cm^{-3}$)	Thickness (cm)	ρ_0 ($g.cm^{-3}$)	Porosity
Agarose	0.0018	0.002468826	1	0.00186	0.729091	27.09
Agarose/PVP	0.0026	0.005123478	1.067	0.00386	0.507468	52.44
Agarose/PVA	0.0022	0.006185339	1.087	0.00466	0.35568	67.28
Agarose/PVA + 0.9PVP	0.0045	0.009901852	1.11	0.00746	0.45446	59.06
Agarose/PVA + 1.8PVP	0.0056	0.01152119	1.127	0.00868	0.486061	56.87
Agarose	0.0017	0.003875792	1	0.00292	0.003875792	56.14
Agarose/PVP	0.0037	0.004486362	1.067	0.00338	0.004486362	22.72
Agarose/PVA	0.0035	0.013538725	1.087	0.0102	0.013538725	76.23
Agarose/PVA + 0.9PVP	0.0036	0.011388457	1.11	0.00858	0.011388457	71.52
Agarose/PVA + 1.8PVP	0.0032	0.011919387	1.127	8.98E-03	0.011919387	76.18

Table S3. Main representative tensile test parameters for agarose membranes from the stress-strain curves shown in Figure S5. *E*: Young´s modulus; σ_y: tensile stress at break; ε_b: elongation at break.

	***E* (MPa)**	**σ_b (MPa)**	**ε_b (%)**
Agarose/PVA in iPrOH	116	10.4	33.9
Agarose/PVA + 1.8PVP in iPrOH	219	12.7	12.6
Agarose/PVA in iPrOH/BuOH	73	5.7	27.5
Agarose/PVA + 1.8PVP in iPrOH/BuOH	150	6.5	6.5

Table S4. Comparison of the electrochemical performance of different separators in a $Na_3V_2(PO_4)_3$/Na half cell.

**Separator**	**Porosity (%)**	**Ionic conductivity ($mS \cdot cm^{-1}$)**	**Capacity ($mAh \cdot g^{-1}$)**	**Reference**
Carboxymethyl cellulose	N.R.	1.72	94 at C/10	1
Polysulfonamide-based	N.R.	1.20	101 at C/5	2
Polypropylene	N.R.	0.36	91 at C/5	2
Chitin nanofiber	54	0.064 (for LIB electrolyte)	~70 at C/10	3
Agarose-based	67	0.72	116 at C/10	This work

References

(1) Casas, X.; Niederberger, M.; Lizundia, E. A Sodium Ion Battery Separator with Reversible Voltage Response Based on Water-Soluble Cellulose Derivatives. *ACS Appl. Mater. Interfaces* **2020**, *12*, 29264–29274.

(2) Zhang, J.; Wen, H.; Yue, L.; Chai, J.; Ma, J.; Hu, P.; Ding, G.; Wang, Q.; Liu, Z.; Cui, G.; Chen, L. In Situ Formation of Polysulfonamide Supported Poly(Ethylene Glycol) Divinyl Ether Based Polymer Electrolyte toward Monolithic Sodium Ion Batteries. *Small* **2017**, *13* (2), 1601530.

(3) Zhang, T.-W.; Shen, B.; Yao, H.-B.; Ma, T.; Lu, L.-L.; Zhou, F.; Yu, S.-H. Prawn Shell Derived Chitin Nanofiber Membranes as Advanced Sustainable Separators for Li/Na-Ion Batteries. *Nano Lett.* **2017**, *17* (8), 4894–4901.
